# Toxicity Studies of Chanoclavine in Mice

**DOI:** 10.3390/toxins11050249

**Published:** 2019-05-02

**Authors:** Sarah C. Finch, John S. Munday, Jan M. Sprosen, Sweta Bhattarai

**Affiliations:** 1 AgResearch Limited, Ruakura Research Centre, Private Bag 3123, Hamilton 3240, New Zealand; jan.sprosen@agresearch.co.nz (J.M.S.); sweta.bhattarai@agresearch.co.nz (S.B.); 2 Institute of Veterinary, Animal and Biomedical Sciences, Massey University, Private Bag 11 222, Palmerston North 4442, New Zealand; J.Munday@massey.ac.nz

**Keywords:** chanoclavine, toxicology, subchronic feeding study, acute toxicity, endophyte, *Epichloë*

## Abstract

*Epichloë* endophytes have been used successfully in pastoral grasses providing protection against insect pests through the expression of secondary metabolites. This approach could be extended to other plant species, such as cereals, reducing reliance on pesticides. To be successful, the selected endophyte must express secondary metabolites that are active against cereal insect pests without any secondary metabolite, which is harmful to animals. Chanoclavine is of interest as it is commonly expressed by endophytes and has potential insecticidal activity. Investigation of possible mammalian toxicity is therefore required. An acute oral toxicity study showed the median lethal dose of chanoclavine to be >2000 mg/kg. This allows it to be classified as category 5 using the globally harmonized system of classification and labelling of chemicals, and category 6.1E using the New Zealand Hazardous Substances and New Organisms (HSNO) hazard classes, the lowest hazard class under both systems of classification. A three-week feeding study was also performed, which showed chanoclavine, at a dose rate of 123.9 mg/kg/day, initially reduced food consumption but was resolved by day seven. No toxicologically significant effects on gross pathology, histology, hematology, or blood chemistry were observed. These experiments showed chanoclavine to be of low toxicity and raised no food safety concerns.

## 1. Introduction

*Epichloë* endophytes are fungal symbionts of cool-season grasses that produce a diverse array of secondary metabolites. One such association, between *Epichloë festuca* var. *lolii* (formerly *Neotyphodium lolii*) and perennial ryegrass (*Lolium perenne*) is essential for the plants survival in New Zealand agricultural conditions [1]. This is due to the expression of secondary metabolites, such as peramine, which protect the plant from insect attack [2,3,4]. Similarly, the association between *E. coenophiala* (formerly *N. coenophialum*) and tall fescue (*Lolium arundinaceum* (Schreb.) Darbysh (syn *Festuca arundinacea*)) is of vital importance to farming systems in the USA because expression of the loline alkaloids confers insect resistance [5,6,7]. Unfortunately, in addition to these beneficial secondary metabolites, endophytes can also produce compounds that cause animal disease. In New Zealand, livestock grazing the naturalised (wild-type or common-toxic) ryegrass–endophyte association can develop the neurological disease ryegrass staggers caused by the compound lolitrem B [8,9]. In the USA, the naturalised fescue–endophyte association is responsible for heat stress of cattle and sheep, fescue-foot syndrome of cattle, as well as animal production issues, which are induced by ergot-alkaloids, including ergovaline [10,11,12]. To harness the beneficial effects of endophytes on insects while minimising the detrimental effects on livestock, research has focused on the screening of endophytes found in other grasses from around the world. Endophytes with a favourable chemical profile can be selected and inoculated into commercial grass cultivars. This method has been successful, and a number of perennial ryegrass and tall fescue products are now commercially available that contain selected endophytes, which provide superior animal safety and pasture persistence and production compared to the naturalised endophyte–grass associations [1].

Because of the success of selected endophyte technology in the pastoral sector, it has been hypothesised that this approach could be used in other plant species such as cereals [13]. Asexual *Epichloë* endophytes have been found to be naturally occurring in Hordeeae grasses, although they are not present in modern cereal grasses. Hordeeae grasses such as wheat (*Triticum aestivum*), barley (*Hordeum vulgare*), and rye (*Secale cereale*) are essential components of both human and domestic animal diets [14], but the current production of these cereal crops is reliant on the use of pesticides. As an alternative, development of endophytic cereals has the potential to provide crops with protection against insect pests with a reduced reliance on synthetic chemicals. However, for this approach to be successful, the selected endophyte must not only provide insect resistance but also be safe for consumption. In our research, a number of fungal endophytes have been isolated from wild relatives of modern cereals (*Elymus* and *Hordeum*) [13]. The targeted endophytes are those that do not express known animal toxins such as lolitrem B and ergovaline, but that do express compounds that are active against insects such as the lolines and peramine. Lolines are not associated with toxicity of grazing animals [15,16]. A short-term toxicity study using mice raised no toxicity issues [17], suggesting that these compounds are an appropriate target.

**Figure 1:**
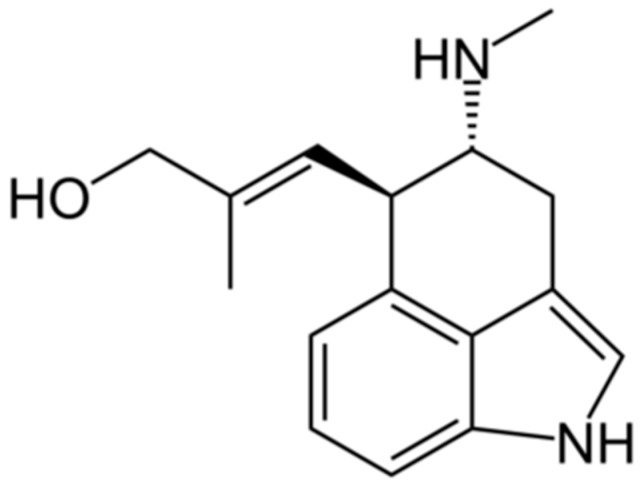
Structure of chanoclavine.

In addition to lolines and peramine, the clavines may be another class of compounds to target for inclusion into cereals. Clavines are the simplest compounds of the ergot-alkaloid class produced by the early steps of the ergot-alkaloid biosynthetic pathway, which then progresses to produce lysergic acid and its amides and ultimately the highly complex ergopeptines such as ergovaline [18]. Some endophytes express only clavine compounds, and others express the whole range of ergot-alkaloid derivatives. It is often found that compounds such as chanoclavine (of the clavine class) can accumulate to be at a similar concentration as the ergopeptine alkaloids. This inefficient flow of intermediates to the biosynthetic end product is unusual, and it has been hypothesised that intermediates, such as chanoclavine, may provide some benefit to the endophyte or to its host plant [19,20]. While it is known that ergovaline has an effect on some insect pests such as the African black beetle (*Heteronychus arator*) [21], research into the effects of chanoclavine (Figure 1) is limited. While initial research suggests that ergot-alkaloids of the clavine class may be less effective in reducing insect damage than the animal-toxic ergopeptine class, the clavines have been shown to deter the feeding of fall armyworm (*Spodoptera frugiperda*) [22]. Ergovaline is well documented to cause fescue toxicosis, which is associated with a dramatic lowering of serum prolactin levels in grazing animals [12]. In vitro testing of ergovaline and chanoclavine on cultured anterior pituitary cells showed that ergovaline lowered prolactin secretion at a concentration of 0.5 nM, whereas an effect of chanoclavine was observed only at a concentration of 1000 nM [23]. This suggests that chanoclavine is unlikely to have a significant effect on serum prolactin levels in grazing animals, and there are no documented reports of animal toxicity induced by chanoclavine.

Further insect testing would be required to determine whether endophytes that produce clavines, such as chanoclavine, should be actively targeted for inclusion into cereal crops. However, if the clavines are found to deter insects, it would be equally important to determine whether these compounds have any mammalian toxicity. Even if it is found that the bioactivity of the clavines is low, information on the toxicity of chanoclavine is important because this compound can be produced by endophytes, which express lolines and peramine without lolitrem B or ergovaline, and are therefore of interest to the cereal endophyte research program. The objective of this study was to provide preliminary information on the toxicity of chanoclavine by performing an oral, acute toxicity determination as well as a three-week subchronic feeding study in mice.

## 2. Results

### 2.1. Acute Toxicity Testing

All five mice dosed with chanoclavine at the limit dose (2000 mg/kg) survived, although some signs of neurotoxicity were observed in three of the five mice. Tremors were detected in two mice, and movement was characterised by splaying of the back legs. Time of onset of these toxic symptoms was between 1 and 5 h post-dosing, but the mice were moving and feeding normally by 24 h. One further mouse exhibited a weak grip 4 h post-dosing, but this was resolved within 2 h. The appearance, behaviour, and growth of all mice remained normal throughout the subsequent 14-day observation period. No abnormalities were observed in any of the animals at necropsy.

Results of the acute oral toxicity testing of chanoclavine indicated that the median lethal dose (LD_50_) was greater than the given dose rate of 2000 mg/kg. In accord with OECD (Organisation of Economic Co-operation and Development) guideline 425, this dose rate was the limit dose such that further dosing at higher rates was not required. A median lethal dose of greater than 2000 mg/kg allowed classification of the oral toxicity of chanoclavine as category 5 using the globally harmonized system of classification and labelling of chemicals (GHS) [24]. Using the New Zealand HSNO hazard classes, this equates to category 6.1E [25].

### 2.2. Short-Term Toxicity Study

#### 2.2.1. Analysis of Mouse Diet

Taking into account moisture content of the prepared diet, the theoretical concentration was 563 µg/g chanoclavine. Analysis of the three diet chunks taken from the prepared discs yielded concentrations of 565 µg/g (100%), 629 µg/g (112%), and 565 µg/g (100%). This indicated that chanoclavine was stable through the diet-making process and was present in a homogenous manner throughout the prepared diet.

#### 2.2.2. Clinical Observations and Appearance

The appearance, movement, and behaviour of all mice remained normal throughout the experimental period.

#### 2.2.3. Bodyweight and Food Consumption

**Figure 2:**
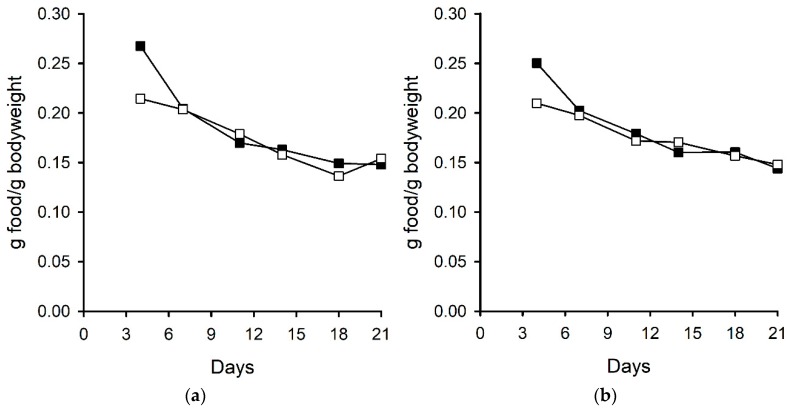
Food consumption of (**a**) male and (**b**) female mice fed control (■) and chanoclavine (□, 563 µg/g) diets.

**Figure 3:**
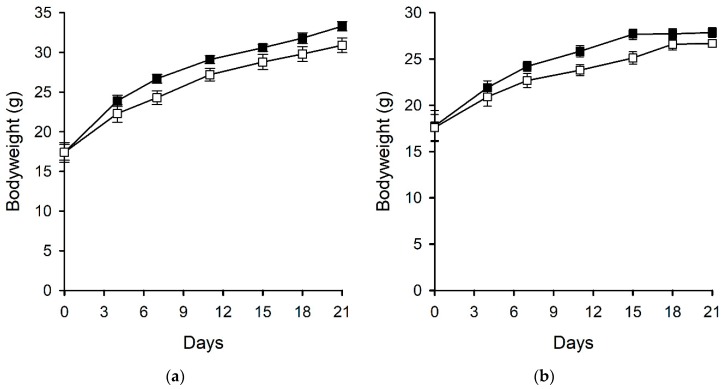
Bodyweights of (**a**) male and (**b**) female mice fed control (■) and chanoclavine (□, 563 µg/g) diets. Error bars represent standard errors of the mean but are too small to be visible in some cases.

Ergovaline is well recognised to reduce food intake in a range of animals including cattle, birds, rabbits, and small mammals [26,27,28]. An experiment using perennial ryegrass-Neotyphodium sp. Lp1 with altered ergot alkaloid profiles (due to the knockout of endophyte genes) showed that endophyte-infected plants, devoid of ergot-alkaloids, were preferred by rabbits over endophyte-free plants. This preference was reduced in plants containing either all of the ergot-alkaloids (ergopeptines and clavines) or in those containing only clavines. However, only those plants containing ergovaline were shown to affect food consumption of rabbits [29]. In the current experiment, which used diets of far higher chanoclavine concentrations than those used by Panaccione (2006) (563 µg/g compared with 1.2 µg/g), food intake of the male and female mice fed a diet containing chanoclavine was lower compared with those fed a control diet in the initial stages of the experiment. However, by day seven, there was no difference in the food intake per gram of bodyweight for mice fed the two different treatment diets, and when expressed in this way, there was no overall treatment effect for the male (*p* = 0.221) or female (*p* = 0.228) mice (Figure 2). This initial food intake difference was reflected in the mouse bodyweights although, overall, there was no treatment effect on the bodyweights of the male (*p* = 0.139) or female (*p* = 0.141) mice (Figure 3). 

#### 2.2.4. Hematological and Serum Biochemical Data

The hematological and serum biochemical data for day 21 are presented in Table 1 and Table 2. The only statistically significant difference observed in the hematological data was a higher neutrophil percentage in the female mice fed a control diet compared with those fed a diet containing chanoclavine. The only statistically significant difference observed in the serum biochemical data of male mice was that the levels of total bilirubin were higher in control mice compared with those fed a diet containing chanoclavine. For the female mice, a statistically significant difference was observed in the concentrations of total protein and albumin, which were higher in mice fed a diet containing chanoclavine compared with those fed a control diet. These differences were not considered to be toxicologically meaningful.

#### 2.2.5. Organ Weights

Absolute and relative organ weights are presented in Table 3 and Table 4. The only statistically significant difference was the absolute and relative weight of the kidneys of the male mice. This difference was not considered to be toxicologically meaningful.

#### 2.2.6. Histological Examination

Examination of tissues from male mice in the control group revealed that one mouse had a single small focus of inflammation within the liver. This focus was not associated with significant hepatocellular injury. Similar foci were also visible within three male mice that were fed chanoclavine. No other histological lesions were observed on examination of tissues from the male mice. Examination of tissues from the control female mice revealed all five mice had scattered foci of inflammation within the hepatic parenchyma. While inflammation was mild and not associated with significant hepatocellular damage in two mice, some of the larger foci in the other three animals contained swollen eosinophilic hepatocytes that demonstrated loss of cell features, consistent with mild hepatocellular damage. Examination of tissues from the female mice that received chanoclavine revealed small foci of inflammation present within the liver of four of the mice. This inflammation was associated with scattered, degenerate hepatocytes in three animals. In addition, one mouse had a small focus of lymphocytes and macrophages within the submucosa of the bladder. No other lesions were observed on examination of tissues from the female mice. The cause of the hepatic changes was uncertain; however, as they developed with approximately equal frequency and severity in the control and treated mice, this change was considered extremely unlikely to be due to chanoclavine toxicity. 

## 3. Discussion

The toxicity of chanoclavine was investigated. Acute toxicity testing showed neurotoxicity in some animals at the limit dose of 2000 mg/kg. This is of no concern, as this extremely high dose rate is not biologically relevant. Since no deaths were observed at this dose rate, the median lethal dose (LD_50_) of chanoclavine is, therefore, greater than 2000 mg/kg. This allowed it to be classified in category 5 using the GHS labelling system and in category 6.1E using the New Zealand HSNO hazard classes. This is the lowest hazard class under both systems of classification. A short-term feeding trial was also performed, whereby a group of male and female mice were fed either a diet containing pure chanoclavine or regular mouse food. Using food consumption and bodyweight data, the dose rate of chanoclavine was calculated for each of the biweekly measurement periods. This showed that the male and female mice ate on average 123.3 and 124.4 mg/kg/day chanoclavine, respectively. In the initial stages of the three-week experiment, mice fed the chanoclavine diet ate less and gained weight more slowly than the mice fed a control diet. However, by day seven, the food consumption of both groups was equivalent on a food intake per gram of bodyweight basis. No toxicologically significant effects were noted in hematological or serum biochemical parameters. Although foci of inflammation were noted in the livers of a number of mice, this was observed in both the control and chanoclavine-fed animals, so it was considered to be unrelated to the treatment diet. No other significant effects were noted on histological examination.

The concentration of chanoclavine that could be present in endophyte-infected cereals is unknown. However, in the acute oral toxicity study, the limit dose of 2000 mg/kg was reached, and in the feeding trial a very high dose rate was chosen. The amounts fed to mice in this study would be equivalent to a 70 kg human consuming 8.6 g of chanoclavine per day. These experiments, therefore, show that chanoclavine is of low toxicity and should be safe for inclusion into cereal crops. However, once a chanoclavine-producing endophyte is successfully inoculated into a cereal crop, further toxicological work will be required.

## 4. Materials and Methods

### 4.1. Chanoclavine

Chanoclavine (>98.69% purity) was purchased from Alfarma s.r.o (Prague, Czech Republic) and was stored at 4 °C before use.

### 4.2. Animals

For acute toxicity testing, Swiss albino mice (5 females, 4–5 weeks old, and 18–20 g) were used. For the short-term toxicity study, Swiss albino mice (10 male and 10 female, 3–4 weeks old, and 14–19 g on day –3) were again used. Animals were housed in groups of five in a temperature-controlled room (21 ± 1 °C) with a 12 h light–dark cycle. They were allowed access to food and water ad libitum. All animal manipulations were approved by the Ruakura Animal Ethics Committee established under the Animal Protection (code of ethical conduct) Regulations Act, 1987 (New Zealand). Acute toxicity: project Number 13242, approval date 1 May 2014; Subchronic toxicity: project Number 13508, approval date2 April 2015.

### 4.3. Acute Toxicity Protocol

Acute toxicity was determined according to the principles of OECD guideline 425 [30] using the limit dose of 2000 mg/kg. Pairs of mice were randomly selected and fasted overnight. Mice were weighed immediately before dosing, and the amount of chanoclavine required was calculated on a mg/kg basis. The required amount of chanoclavine was added into the glass pipette tip of a positive displacement pipette along with a small portion of ground mouse food (20 mg) and water (50 µL) to yield a paste. This paste was applied over the tongue of the mouse, with care taken to ensure that the entire dose was swallowed. Control mice were dosed in an analogous manner with a ground mouse food (20 mg) and water (50 µL) mixture. To avoid diurnal variations in response, all dosing was conducted between 9.30 and 10.00 am. The mice were monitored intensively during the day of dosing and then examined and weighed each day for a further 14 d, after which they were killed by CO_2_ inhalation and necropsied. In total, five pairs of mice were dosed.

### 4.4. Short-Term Toxicity Study

Mouse diets were prepared from Teklad Global 2016 mouse food pellets (Harlan UK, Bicester, England), which were finely ground using a Udy cyclone sample mill (Udy Corporation, Fort Collins, CO, USA). Initially, a test batch of chanoclavine diet was prepared to check stability and diet homogeneity by mixing chanoclavine (29.8 mg) with ground mouse food (50 g) using a cake mixer, adding water (45 mL), and drying as described below. Three separate samples of diet were taken for analysis and ground using a mortar and pestle. The diets for the feeding study were prepared by mixing chanoclavine (141.6 mg) with ground mouse food (200 g) using a cake mixer. Water (90 mL) was added to 100 g aliquots of this mixture and combined to form a paste. This was split evenly into 14 portions, which were formed into discs and dried in a fan oven (50 °C, 24 h). This resulting diet had a moisture content of 11.3% and a chanoclavine concentration of 628 µg/g (wet weight). Control diets were prepared in an analogous manner to yield diets with a moisture content of 13.8%. Diets were prepared twice weekly during the experimental period. 

Mice were randomly assigned to their cage groups 3 d before the start of the experiment. They were fed prepared control diets so that they could get used to the new form of their food. Each group was comprised of five female and five male mice (housed by gender); group 1 was the control group, and group 2 was the chanoclavine group. During the experiment, food consumption of each cage group of five mice was measured every second day. At this time, the appearance, movement, and behaviour of each mouse was observed to check for any changes. Biweekly the body weight of each mouse was measured. Food consumption of each cage group of mice was calculated from the quantity of diet eaten by the five mice divided by the total bodyweight. At the conclusion of the three-week experiment, all mice were killed by CO_2_ inhalation, and a blood sample was collected by heart puncture using heparin as the anticoagulant. Whole blood was analysed to measure hematocrit values (HCT), hemoglobin levels (HB), mean corpuscular volumes (MCVs), mean corpuscular hemoglobin (MCH), mean corpuscular hemoglobin concentrations (MCHCs), and red and white cell counts, while activities of alanine aminotransferase (ALT), aspartate aminotransferase (AST), and creatine kinase (CK) as well as concentrations of bilirubin, total protein (TP), albumin (ALB), globulin, sodium, potassium, chloride, and creatinine (CRN) were determined in plasma (New Zealand Veterinary Pathology, Hamilton, New Zealand). Necropsies of all mice were performed to check for any macroscopic changes, and the weights of brain, heart, kidneys, liver, and spleen were measured and expressed as a percentage of body weight for each mouse. These organs, along with adrenals, lungs, pancreas, gastrocnemius, jejunum (3 mm section), ovary and uterus or testes, spinal cord (3 × 2 mm sections), stomach (washed), thymus, and urinary bladder were placed in 4% buffered formaldehyde to allow fixation before being routinely processed for histology. The same pathologist examined all samples and was unaware of the treatment groups.

### 4.5. Measurement of Chanoclavine in Mouse Diet

One of the prepared chanoclavine diets was taken after the drying process, and three samples were taken from different areas of the disc. These samples were ground using a mortar and pestle. Aliquots (50 mg) were extracted with 50% methanol (1 mL) containing an internal standard (festuclavine, 0.2 µg/mL). Filtered extracts were analysed according to the method of Rasmussen et al. [31].

### 4.6. Statistical Analysis

Bodyweight, food intake, hematology, serum biochemistry, and organ weight data from the short-term toxicity study were analysed by one-way ANOVA followed by Fisher’s protected least significant difference test at the 5% significance level. Data from each measurement time, and for each gender, were analysed separately. Since the genders for each treatment group were housed together, there was no gender replication of the treatments in the trial, so the assumption was made that mice within a cage behaved independently of one another. Boxplots and residual plots were inspected for departures from normality and homogeneity of variance. All analyses were conducted in GenStat version 16 (VSN International, Hemel Hempstead, UK).

## Figures and Tables

**Table 1:** Hematology data from mice fed treatment diets for 21 d.

Item	Control ^1^	Chanoclavine ^1^	SED	*p*-Value
*Males*				
HCT (L/L)	0.46 ± 0.01	0.47 ± 0.01	0.01	0.620
HB (g/L)	140.8 ± 3.6	142.8 ± 4.9	2.72	0.483
RBC (×10^12^/L)	8.8 ± 0.30	8.9 ± 0.4	0.21	0.527
MCV (fL)	52.8 ± 1.5	53.2 ± 1.3	0.88	0.663
MCH (pg)	16.2 ± 0.4	16.2 ± 0.4	0.28	1.000
MCHC (g/L)	303.8 ± 2.6	303.6 ± 4.6	2.35	0.934
WBC (×10^9^/L)	10.3 ± 1.6	10.4 ± 1.7	1.06	0.884
Neutrophil (%)	17.6 ± 1.5	19.4 ± 7.5	3.42	0.613
Lymphocyte (%)	75.8 ± 5.2	69.6 ± 8.9	4.62	0.216
Monocyte (%)	3.2 ± 1.6	4.8 ± 1.5	0.99	0.145
Eosinophil (%)	3.0 ± 2.0	3.0 ± 1.9	1.23	1.000
Basophil (%)	0.4 ± 0.6	3.2 ± 7.2	3.21	0.408
*Females*				
HCT (L/L)	0.47 ± 0.01	0.49 ± 0.04	0.02	0.213
HB (g/L)	145.4 ± 1.3	151.0 ± 12.5	5.61	0.347
RBC (×10^12^/L)	8.9 ± 0.1	9.4 ± 0.8	0.35	0.149
MCV (fL)	52.8 ± 0.8	51.8 ± 1.3	0.69	0.187
MCH (pg)	16.4 ± 0.5	16.0 ± 0.0	0.25	0.141
MCHC (g/L)	311.0 ± 3.7	308.6 ± 3.0	2.14	0.294
WBC (×10^9^/L)	7.4 ± 2.1	8.5 ± 1.9	1.27	0.405
Neutrophil (%)	13.6 ± 3.1 ^a^	9.4 ± 1.7 ^b^	1.59	0.029
Lymphocyte (%)	78.0 ± 3.7	81.4 ± 2.4	1.97	0.122
Monocyte (%)	3.6 ± 1.1	4.0 ± 1.6	0.87	0.659
Eosinophil (%)	4.8 ± 1.3	4.6 ± 0.9	0.71	0.784
Basophil (%)	0.0 ± 0.0	0.6 ± 1.3	0.60	0.347

^1^ Values are means ± standard deviation (*n* = 5). Fisher’s least significant difference was used to compare treatment means. Two means that have no letter in common are statistically different at the 5% level. If no letters are given, the overall *p*-value was not statistically significant at the 5% level. HCT, hematocrit value; HB, hemoglobin level; MCV, mean corpuscular volume; MCHC, mean corpuscular hemoglobin concentration.

**Table 2:** Serum biochemical data from mice fed treatment diets for 21 d.

Item	Control ^1^	Chanoclavine ^1^	SED	*p*-Value
*Males*				
CK (IU/L)	294 ± 69	437 ± 206	91.6	0.164
AST (IU/L)	75 ± 15	75 ± 20	11.1	0.972
ALT (IU/L)	37 ± 12	35 ± 6	6.0	0.748
T.Bil (µmol/L)^2^	1.8 ± 0.4 ^a^	0.9 ± 0.2 ^b^	0.2	0.004
TP (g/L)	54.0 ± 2.2	53.8 ± 2.0	1.4	0.886
ALB (g/L)	33.2 ± 0.8	33.0 ± 1.4	0.7	0.786
Globulin (g/L)	20.8 ± 2.9	20.0 ± 1.4	1.5	0.616
A/G	1.622 ± 0.230	1.660 ± 0.178	0.1	0.787
CRN (µmol/L)	14.0 ± 2.8	17.0 ± 5.8	2.9	0.328
Na (mmol/L)	146 ± 2	146 ± 1	0.9	0.829
K (mmol/L)	15.0 ± 1.4	15.3 ± 1.3	0.9	0.700
Cl (mmol/L)	103 ± 1	105 ± 1	0.6	0.101
*Females*				
CK (IU/L)	569 ± 210	594 ± 209	132.6	0.851
AST (IU/L)	110 ± 20	121 ± 17	11.7	0.366
ALT (IU/L)	31 ± 5	39 ± 9	4.5	0.126
T.Bil (µmol/L) ^2^	1.5 ± 0.7	0.9 ± 0.2	0.3	0.108
TP (g/L)	54.8 ± 1.1 ^b^	57.8 ± 2.3 ^a^	1.1	0.029
ALB (g/L)	36.6 ± 1.1 ^b^	38.8 ± 1.5 ^a^	0.8	0.030
Globulin (g/L)	18.2 ± 0.8	19.0 ± 1.6	0.8	0.347
A/G	2.015 ± 0.140	2.053 ± 0.181	0.1	0.721
CRN (µmol/L)	17.0 ± 0.7	16.8 ± 3.7	1.7	0.908
Na (mmol/L)	148 ± 1	148 ± 4	1.8	0.913
K (mmol/L)	12.4 ± 1.0	12.8 ± 2.1	1.0	0.682
Cl (mmol/L)	106 ± 1	106 ± 2	0.9	0.838

^1^ Values are means ± standard deviation (*n* = 5). Fisher’s least significant difference was used to compare treatment means. Two means that have no letter in common are statistically different at the 5% level. If no letters are given, the overall *p*-value was not statistically significant at the 5% level. ^2^ When total bilirubin (T. Bil) was below the detection limit it was assigned a value of half the detection limit (0.5 µmol/L). CK, creatine kinase; AST, aspartate aminotransferase; ALT, alanine aminotransferase; TP, total protein; ALB, albumin; CRN, creatinine.

**Table 3:** Absolute organ weight data (g) from mice fed treatment diets for 21 d.

	Brain ^1^	Heart ^1^	Kidneys ^1^	Liver ^1^	Spleen ^1^
*Males*					
Control	0.467 ± 0.017	0.181 ± 0.011	0.512 ± 0.019 ^a^	1.832 ± 0.129	0.135 ± 0.029
Chanoclavine	0.453 ± 0.021	0.176 ± 0.019	0.441 ± 0.033 ^b^	1.656 ± 0.183	0.126 ± 0.017
SED	0.012	0.010	0.017	0.100	0.015
*p*-value	0.269	0.623	0.003	0.117	0.579
*Females*					
Control	0.457 ± 0.020	0.167 ± 0.012	0.355 ± 0.017	1.384 ± 0.203	0.146 ± 0.028
Chanoclavine	0.466 ± 0.010	0.155 ± 0.006	0.328 ± 0.029	1.280 ± 0.093	0.132 ± 0.020
SED	0.010	0.006	0.015	0.100	0.016
*p*-value	0.385	0.086	0.117	0.328	0.420

^1^ Values are means ± standard deviation (*n* = 5). Fisher’s least significant difference was used to compare treatment means. Two means that have no letter in common are statistically different at the 5% level. If no letters are given, the overall *p*-value was not statistically significant at the 5% level.

**Table 4:** Relative organ weight data (% of bodyweight) from mice fed treatment diets for 21 d.

	Brain ^1^	Heart ^1^	Kidneys ^1^	Liver ^1^	Spleen ^1^
*Males*					
Control	1.439 ± 0.039	0.559 ± 0.041	1.578 ± 0.079 ^a^	5.638 ± 0.259	0.417 ± 0.097
Chanoclavine	1.500 ± 0.110	0.586 ± 0.090	1.456 ± 0.023 ^b^	5.458 ± 0.251	0.417 ± 0.046
SED	0.052	0.044	0.037	0.161	0.048
*p*-value	0.280	0.555	0.011	0.298	0.999
*Females*					
Control	1.671 ± 0.136	0.610 ± 0.037	1.296 ± 0.059	5.030 ± 0.510	0.530 ± 0.090
Chanoclavine	1.775 ± 0.049	0.592 ± 0.031	1.250 ± 0.101	4.873 ± 0.322	0.504 ± 0.074
SED	0.065	0.022	0.052	0.270	0.052
*p*-value	0.147	0.439	0.404	0.578	0.626

^1^ Values are means ± standard deviation (*n* = 5). Fisher’s least significant difference was used to compare treatment means. Two means that have no letter in common are statistically different at the 5% level. If no letters are given, the overall *p*-value was not statistically significant at the 5% level.
